# Role of Maternal Dietary Protein and Amino Acids on Fetal Programming, Early Neonatal Development, and Lactation in Swine

**DOI:** 10.3390/ani9010019

**Published:** 2019-01-13

**Authors:** Shihai Zhang, Jinghui Heng, Hanqing Song, Yufeng Zhang, Xiaofeng Lin, Min Tian, Fang Chen, Wutai Guan

**Affiliations:** 1 Guangdong Province Key Laboratory of Animal Nutrition Control, College of Animal Science, South China Agricultural University, Guangzhou 510642, China; zhangshihai@scau.edu.cn (S.Z.); Hengjinghui@stu.scau.edu.cn (J.H.); hqsong@stu.scau.edu.cn (H.S.); zhangyufeng@stu.scau.edu.cn (Y.Z.); xiaofenglin@stu.scau.edu.cn (X.L.); tianmin@stu.scau.edu.cn (M.T.); chenfang1111@scau.edu.cn (F.C.); 2 College of Animal Science and National Engineering Research Center for Breeding Swine Industry, South China Agricultural University, Guangzhou 510642, China; 3 College of Animal Science, South China Agricultural University, Wushan Avenue, Tianhe District, Guangzhou 510642, China

**Keywords:** sows, dietary protein level, lactation, fetus development

## Abstract

**Simple Summary:**

Dietary protein is an important nutrient source for sows, necessary for not only growth and production, but also other physiological functions. Protein limitations in maternal diets have the potential to impair fetal myogenesis, while excess maternal dietary protein appears to only have minor effects on early fetal muscle formation. Effects of maternal protein deficiency on increased fat deposition in porcine neonates is inconsistent with gene expressions in the neonates. Sufficient maternal dietary protein can enhance porcine milk protein and fat concentration. Understanding the function of protein and amino acids in sows and the effects on their offspring can provide rational approaches for the regulation of piglet growth and further improvements in meat quality in the future.

**Abstract:**

Maternal nutrition plays a vital role in fetal development, early development of neonates, and lactation and regulates the lifetime productivity of offspring. During pregnancy, maternal nutrition alters expression of the fetal genome and the development of tissues and organs via fetal programming. After parturition, maternal nutrition continues to regulate growth and development of piglets through maternal milk, which contains carbohydrates, lipids, proteins and oligosaccharides. Thus, deficiencies in maternal nutrition are detrimental to development of piglets, which can lead to inefficient growth and decreased carcass merit. Protein is an important nutritional component for sows, which not only functions in muscle development, but also plays a vital role in embryonic and neonatal development and lactation. Although effects of maternal undernutrition on neonatal development have been widely studied in sows, the function of different maternal dietary protein levels on fetal development, neonatal growth and lactation performance of sows is largely unknown. Determination of the effects and underlying mechanisms of maternal dietary protein levels on development of piglets is vital to the pork industry. Therefore, we summarized recent reports regarding mechanisms of effects of maternal protein levels on regulation of conceptus growth and early postnatal development though uterine fetal programming and lactation in swine.

## 1. Introduction

Maternal nutritional excess or deficiency during or after pregnancy can significantly impact the development of offspring. Sufficient maternal nutrition enhances placental growth, vascular development, and placental nutrient transport [1]. Additionally, maternal nutrient supply, possibly acting as a stimulus, contributes to the programming of fetal development and can have permanent effects on anatomy, physiology, and metabolism [2]. Around 14 to 18 days after ovulation and fertilization, the porcine embryo initiates attachment in the uterus [3]. After attachment, the fetus is dependent on maternal nutrition for growth and development. During this period fetal programming occurs, initiated by nutritional and environmental stimulus or insult, and results in changes in organ differentiation, growth, or development, especially muscle and adipose tissue [4]. In meat animals, development of muscle mass is one of the most critical commercial objectives. Skeletal muscle fiber hyperplasia is a determining factor in muscle mass formation. Muscle fibers starts developing in the fetal stage and the number of muscle fibers is fixed at birth in pigs [5]. The growth in muscle mass after birth is then a function of muscle fiber hypertrophy by increasing the fiber diameter and length [6]. Therefore, to insure maximum muscle fiber hyperplasia, sufficient nutrition during pregnancy is critical for efficient livestock production. 

**Figure 1:**
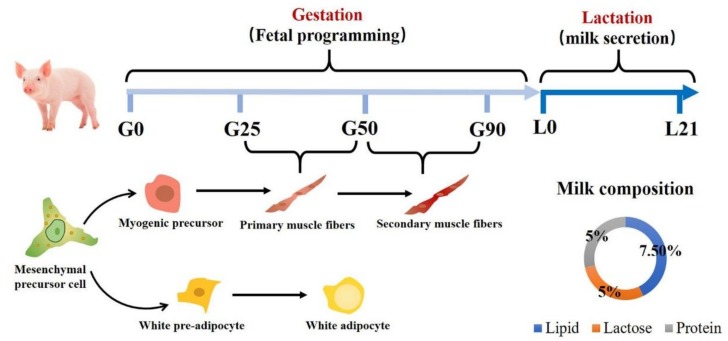
Maternal regulation of early development of neonates through fetal programming and lactation.

Immediately after parturition, porcine milk is the sole nutrient source for growth of neonates and contains 5% lactose, 5% protein, and 7.5% fat [7] (Figure 1). During lactation, sufficient maternal nutrition is required for mammary development, milk volume, and milk quality. In addition, colostrum provides immunoglobulins, the transmission of which is important and essential for piglets to receive passive immunity in the early neonatal period [8]. Sufficient milk intake can enhance gastrointestinal tract development [9,10] and stimulate visceral organ and skeletal muscle protein synthesis [11]. 

Protein, as one of the major macronutrients in diets, has been extensively evaluated for its effect on porcine growth and development. Limited maternal dietary protein can influence fetal and neonatal growth through insufficient and unbalanced amino acids available to the fetuses and neonates [12]. However, there is limited information on the effects of maternal protein and amino acid levels on fetal and early neonatal development through fetal programming and lactation performance of sows. Thus, the objective of this review is to describe the mechanisms whereby maternal protein levels during gestation and lactation regulate conceptus growth and early postnatal development. 

## 2. Muscle and Adipose Tissue Formation of Porcine Fetus 

Fetal programming is closely associated with prenatal and postnatal animal growth and development in livestock [13,14]. Muscle fibers initiate development in the embryonic stage with the number of fibers fixed at birth in pigs [5]. As shown in Figure 1, the time for primary muscle formation is from d 25 to d 50 of gestation, and secondary muscle is formed between d 50 to d 90 of gestation [15]. Secondary fibers are located around primary myotubes using them as a scaffold. The growth of muscle after birth is solely through an increase in muscle fiber size with no formation of new muscle fibers [16]. Nutrient-restricted maternal diets significantly decrease the numbers of muscle fibers in neonatal pigs [17]. Piglets with reduced fiber numbers grow more slowly than normal piglets and tend to have larger muscle fibers and poorer meat quality [17,18]. 

Adipocytes and myocytes are derived from the same mesenchymal precursor cells [19]. Most mesenchymal precursor cells develop into myogenic cells with fewer differentiating into adipocytes (Figure 1) [20]. When muscle formation is restricted, mesenchymal precursor cells tend to form fat and connective tissue [21]. During porcine fetal stages, lipid deposition mainly occurs within muscle fibers [22]. The smallest porcine fetuses have been reported to have higher levels of fat in muscle compared with largest ones [16]. The difference of fat present in muscles can be seen as early as 60 days of gestation. These perturbations in fetal development have a potential influence on meat quality, including meat tenderness.

## 3. Maternal Protein Level on Fetal Skeletal Muscle Development

Although the effects of undernutrition on fetal growth have been extensively studied in sheep [23,24], cattle [25], and swine [17], research on the function of dietary protein on fetal programing has only recently been undertaken. In sows, a protein limited maternal diet (crude protein (CP) = 6.5 %) resulted in a 15% fetal growth retardation [26]. This diet also impaired prenatal myofiber formation, reduced lean meat percentage and resulted in greater fat deposition in offspring [27]. In addition, a low maternal protein diet (CP = 6.5%) was demonstrated to decrease longissimus dorsi muscle weight and its cross-sectional areas at weaning [28]. Muscular fiber number is an important determinant of muscle mass in the pig and is primarily formed during gestation [29]. Protein limited maternal diets have been shown to have negative effects on myofiber formation. Rehfeldt and colleagues (2012) [30] found that a low maternal protein diet (CP = 6.5%) adversely affected myogenesis and muscular differentiation, resulting in lesser primary and secondary myofibers of newborn piglets. A low maternal protein diet also resulted in reduction of muscle fiber number and density in rats [31]. However, Kalbe and colleagues (2017) [32] reported low maternal protein diets (CP = 6.5%) had marginal effects on total number of primary and secondary myofibers of 64 day and 94 day porcine fetuses. Evidence from the literature suggests that limited maternal diets have potential to impair fetal myogenesis. Recent studies on the effects of different maternal protein levels on early fetal muscle development are listed in Table 1.

**Figure 2:**
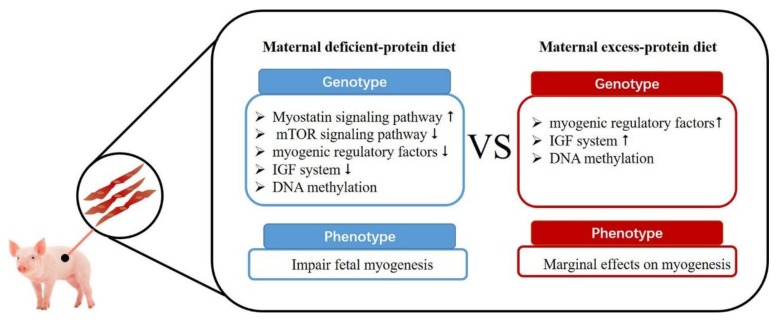
Effects of deficient and excess maternal protein level on fetal fiber formation. Upward-pointing arrow indicates “increase” and downward-pointing arrow indicates “decrease”.

Potential underlying mechanisms for effects of maternal dietary protein levels on fetal muscle development are listed as follows (Figure 2). Firstly, maternal low-protein diets can increase myostatin signaling and inhibit mTOR signaling pathways in the fetus [28,35]. As a member of the TGF-β family, myostatin is an important autocrine/paracrine inhibitor of skeletal muscle growth, preventing muscle precursor cell proliferation and negatively regulating myogenic differentiation [36,37]. The upregulation of myostatin is partly due to the binding of both forkhead box class O family member protein 3 (FoxO3) and glucocorticoid receptor (GR) to the myostatin gene promoter [38]. Maternal low-protein maternal diets could inhibit the mTOR signaling pathway and its downstream targets S6K1 and 4EBP1/eIF4E, a classic signaling pathway for protein synthesis [28]. Secondly, maternal low-protein diets might decrease the myogenic regulatory factors (MRFs) (e.g., myogenin, MRF4) [32]. Myogenic regulatory factors play a vital role in muscle growth and development, and associated genes are considered as candidate genes for lean meat production in pigs [39]. Kablar and colleagues (2013) [21] found that when Myf5 and MyoD were knocked out, progeny deposited excessive amounts of adipose tissue in lieu of muscle. However, maternal protein restriction regulated skeletal muscle changes without modifying myogenic expression in the offspring of rats [40]. Thirdly, maternal protein-deficient diets decrease the expression of the insulin-like growth factor (IGF) system (IGF1, insulin-like growth factor I receptor (IGF1R)) in fetuses [32]. IGFs and insulin-like growth factor-binding proteins (IGFBPs) are essential growth factors for skeletal muscle development [41]. Sow dietary protein might regulate the nutrient supply to the fetus, which further regulates IGFs and IGFBPs produced in the fetus. Similar phenomena have also been observed in cattle [42] and sheep [43]. Fourthly, maternal dietary protein can regulate fiber formation though DNA methylation (DNA methyltransferase 1 (DNMT1), DNMT3a and DNMT3b) [34]. DNA methylation is a critical epigenetic modification in mammals and plays a vital role in muscle development [44]. DNA methylation mainly occurs on a cytosine in a CpG dinucleotide with DNA methyltransferases (DNMTs) [45]. Similarly, maternal protein-restricted diets during pregnancy have been reported to regulate DNA methyltransferase during pregnancy in rats [46] and sheep [47]. Since skeletal muscle plays a vital role in the regulation of metabolic homeostasis, the limited formation and size of skeletal muscle in piglets from dams on protein-deficient diets might contribute to dysfunction of metabolism and reduction of meat quality in the finishing period.

Compared with large numbers of studies on maternal protein-limited diets, limited reports are available on the effect of maternal excess-protein diets on the development of fetal muscle and deposition of adipose tissue. Rehfeldt and colleagues (2012) [30] observed that a maternal high-protein (CP = 30%) diet did not enhance myogenesis and muscular differentiation in the progeny. Rehfeldt and colleagues (2012) [27] reported that a maternal excess-protein diet (CP = 30%) had little effect on the fetal programming of progeny muscle and adipose tissue deposition. Kalbe and colleagues (2017) [32] observed high maternal protein diets (CP = 30%) had marginal effects on body composition and total number of primary and secondary myofibers of 64 day and 94 day fetuses. Therefore, compared with protein-limited maternal diets, which have detrimental effects on muscular development, excess-protein maternal diets seem only to have minor effects on early fetal muscle formation. However, maternal high-protein diets still positively regulate expression of a large number of genes in porcine fetal muscle (Figure 2). Excess maternal protein can: (1) increase myogenic regulatory factors (MYOD, MYOG) [32]; (2) enhance the IGF system (IGF1R, and IGFBP5) [32]; and (3) regulate DNA methylation (DNMT1, DNMT3a and DNMT3b) [34]. The regulation of the expression of large numbers of genes in muscle through maternal protein levels indicates that potential changes in muscle characteristics in growing pigs are possible. However, limited reports are available on the effect of maternal excess dietary protein on characteristics of meat (muscle growth and adipose deposition) from progeny in the finishing period. More research is needed to clarify effects of maternal excess dietary protein on progeny growth and development.

## 4. Maternal Protein Level on Adipose Tissue Development

A number of experiments indicated that a maternal nutrient-restricted diet can result in adult obesity in the offspring of humans and rats with increased blood cholesterol and triacylglycerol (TG) levels [48,49]. However, protein-limited maternal diets during gestation may not consistently increase adipose tissues in neonatal pigs. Pan and colleagues (2018) [33] found a protein-limited maternal diet (CP = 7.5%) decreased the backfat thickness and the subcutaneous fat TG concentration in weaning pigs. Similarly, Rehfeldt and colleagues (2012) [30] observed that subcutaneous fat and perirenal fat were decreased in the offspring when sows were fed a low-protein diet (CP = 6.5%). 

**Figure 3:**
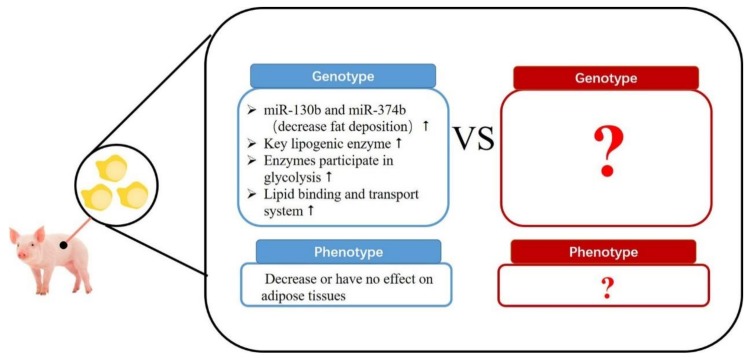
Effects of deficient and excess maternal protein level on neonate fat formation. Upward-pointing arrow indicates “increase” and downward-pointing arrow indicates “decrease”.

Effects of maternal protein-restricted diets on adipose tissue in the progeny is still uncertain (Figure 3). MicroRNAs (miRNAs) regulate post-transcription by promoting targeted RNA degradation and translational arrest [50]. Recently, research indicated that sows fed a low-protein diet upregulated miR-130b (targeting the PPAR-γ 3′-untranslated region (UTR)) and miR-374b (targeting the C/EBP-β 3′-UTR), which might inhibit early lipid deposition in offspring [51]. A proteome study compared gene expression of fat adipose tissue from piglets in a maternal low-protein diet treatment versus a maternal normal-protein diet treatment, and found that gene expressions were not consistent with phenotype [52]. The level of the key lipogenic enzyme, fatty acid synthase (FAS), is highly expressed in subcutaneous fat of piglets from maternal low-protein diet treatments, which might promote fat deposition in subsequent growing periods [53]. Enzymes that participate in glycolysis (e.g., aldolase, enolase 1, and pyruvate dehydrogenase) are also up-regulated in maternal low-protein diet piglets. Furthermore, a maternal protein-restricted diet also appears to increase the lipid binding and transport system in piglet adipose tissue by enhancing intercellular lipid transport in offspring from sows on protein-deficient diets [53]. Up-regulation of these functional genes might contribute to fat accumulation in the finishing period. Currently, information regarding gene expression of fetal adipose tissue as affected by maternal dietary protein is still very limited and requires further study. There is evidence that a maternal low-protein diet regulates the appetite of offspring and initiates a preference for high fat food, which can also lead to obesity [54,55]. However, since fat content of diets are regulated in commercial pig production, this is less relevant to adipose deposition in finishing pigs.

Obesity appears to be closely related to malfunctioning of brown fat [56]. In sheep, maternal protein-restricted diets may result in adult obesity in their offspring, which might be caused by the development of adipose tissue mitochondria (brown adipose tissue-specific uncoupling protein 2 (UCP2) and peroxisome proliferator-activated receptors (PPAR)α) [57,58]. However, Dauncey and colleagues (1981) [59] found only small quantities of brown adipose tissue in pigs and Trayhurn and colleagues (1989) [60] reported that pigs do not contain brown adipose tissues but only white adipose tissue. This information indicates that protein-limited maternal diets in swine might not regulate adipose tissue deposition in offspring through adipose tissue mitochondria.

## 5. Maternal Protein Level on Sow Lactation

As the sole nutrient source for neonates, sufficient milk quantity and quality are essential for growth and development of piglets [61]. Milk synthesis and secretion are largely regulated by nutrients in the maternal diet. The nutrients for the synthesis of milk protein are derived from blood amino acids, which originate from dietary amino acids and body reserves. Dietary protein levels are a critical factor in milk protein synthesis. Højgaard and colleagues (2018) [62] conducted an experiment with 594 sows allocated to one of six diets with standardized ileal digestible (SID) CP of 96, 110, 119, 128, 137, and 152 g/kg. This experiment found milk yield and milk fat reached a peak at 128 g and 110 g dietary SID protein/kg, respectively, while milk protein linearly increased from 4.1% to 5.1% with the increase of the dietary protein level. Similar research conducted with different SID CP levels of 104.3, 113.3, 120.9, 128.5, 139.2 or 150.0 g/kg in 544 sows found milk CP reach to its maximum (5%) when sows were fed with 136 g SID CP/kg [63]. Furthermore, milk fat linearly increased when more protein was supplemented in the diet. Dissimilar to milk protein and fat, lactose does not appear to be affected by maternal dietary protein level [64]. These studies indicated that maternal excess-protein diets have a detrimental effect on milk composition. Our hypothesis is further supported by Manjarin and colleagues (2018) [65] who reported that reducing CP in lactating diets from 16.0% to 13.2% increased milk yield and milk protein percentage. Appropriate concentrations of blood amino acids support functional metabolic processes including production of nitric oxide, polyamines, glutathione, taurine, thyroid hormones, and serotonin, while excess amino acids metabolize into ammonia, homocysteine, and asymmetric dimethylarginine and have detrimental effects on sows [66]. Additionally, restricted dietary CP with improved dietary AA balance could increase the uptake of limiting AA and enhance the efficiency of dietary N and AA utilization by the mammary gland [67]. In contrast, Jang and colleagues (2014) [68] found no differences were observed in milk composition during lactation when sows were fed 11%, 13%, 15%, or 17% CP diets. Renaudeau and Noblet [69] reported decreasing dietary protein from 17.6% to 14.2% did not affect milk protein level. These contradictory results might be due to the limited numbers of sows in experiments of Jang and colleagues (2014) [68] and Renaudeau and Noblet [69]. 

## 6. Functional Amino Acids and Mammary Gland Development and Lactation

Mammary gland growth continues during lactation with associated increases in DNA and tissue protein [70]. The uptake of arginine, leucine, isoleucine, valine, phenylalanine, and threonine by the mammary gland exceeds their output in milk, indicating important roles of these amino acids in mammary gland development [71]. 

Arginine is considered an essential amino acid in regulation of the mammary gland and in secretion of milk. It is a substrate for the synthesis of NO and polyamines, both of which are important for facilitating vascular growth and blood flow in the mammary gland [72]. Furthermore, arginine enhances the concentrations of plasma insulin and growth hormone in sows and piglets [73,74]. More than 90% of arginine is converted into proline (46%), ornithine (31%), and urea (17%) in the mammary gland, while only a small amount of arginine is converted into NO [75]. Recently, L-arginine was found to increase protein synthesis in porcine mammary epithelial cells with the activation of the mTOR signaling pathway [76]. Even though L-arginine appears to have potential roles in milk synthesis in in vitro models, the effects of dietary supplementation of arginine on the sow is still controversial. Holanda and colleagues (2018) [77] reported dietary supplementation of 1% Arg (final concentration 2.15%) to lactating sows increased blood vessel numbers and diameter in the mammary gland. However, most current reports found L-arginine to have limited effect on mammary gland function. Krogh and colleagues (2016) [78] reported daily supplementation with Arg did not affect the yield and composition of mature milk, and only had potential to increase the protein level in colostrum, possibly due to upregulation of IgG concentration. Krogh and colleagues (2017) [79] found that daily supplementation with 25 g/d Arg (final concentration 2.11%) did not increase mammary plasma flow or mammary uptake of AA. Furthermore, Bass and colleagues (2017) [80] reported that supplementation of sows with 44 g SID ARG/d did not improve piglet birth weight or litter performance, and even tended to decrease the individual birth weights. Future studies are needed to clarify the most effective time of supplementation and optimal dose of L-arginine for sows. 

Branched chain amino acids (BCAAs) are also catabolized in the mammary gland and converted into glutamine and aspartate [81]. Isoleucine and leucine play a vital role in protein synthesis in the mammary gland [82,83]. Rezaei (2015) [84] reported that increasing the extracellular concentrations of BCAA from 0.1 to 2 mM enhanced the rate of protein synthesis and cell proliferation with the activation of the mTOR signaling pathway in porcine mammary epithelial cells. Although little information is available on effects of different dietary leucine levels on milk secretion of sows, studies regarding the leucine metabolite *β*-hydroxy-*β*-methyl butyrate (HMB) have been published. Nissen and colleagues (1994) [85] reported that supplementation of HMB in the diet increased fat content of sow milk. Similarly, Flummer C and Theil PK [86] found HMB increased milk content of fat, dry matter and energy in sows. In in vivo experiments, increasing the dietary SID valine:lysine level from 0.63 to 1.03 enhanced feed intake and levels of analyzed amino acids in sow colostrum [87]. However, Strathe and colleagues (2017) [88] found that increasing the dietary valine-to-lysine ratio (from 0.84 to 0.99) had no effect on feed intake, milk production, or milk composition of sows. These results indicate that deficiencies in valine can be detrimental to milk secretion. Because valine cannot activate the mTOR signaling pathway as can leucine and isoleucine, enhancement of amino acid concentrations in milk associated with valine supplementation might be caused by upregulation of feed intake. Richert and colleagues (1997) [89] found dietary supplementation of isoleucine increased milk DM, CP, and fat. Since supplementation of any BCAA could lead to unbalance in the other two BCAAs, supplementation of the three BCAA in combination has been done [90]. The effects of a dietary BCAA combination on lactation function in sows is still controversial. Dunshea and collegaues (2005) [91] found dietary BCAA content can increase milk protein secretion. However, Appuhamy and colleagues (2011) [92] found infusion of BCAA stimulated synthesis of body protein in sows, but had no apparent benefits in milk protein synthesis. Moser and colleagues (2000) [93] reported that increasing dietary valine (from 0.80% to 1.20%), isoleucine (from 0.68% to 1.08%) or leucine (from 1.57% to 1.97%) had no effect on milk composition. Currently, studies regarding different dietary levels and ratios of leucine, isoleucine and valine on sow milk composition are still very limited and additional research is warranted. 

## 7. Conclusions and Future Perspectives

Dietary protein is an important nutrient source for sows, necessary for not only growth and production, but also for other physiological functions. Protein limitations in maternal diets have the potential to impair fetal myogenesis through a number of mechanisms: (1) increasing myostatin signaling and inhibiting mTOR signaling pathways; (2) decreasing myogenic regulatory factors; (3) down-regulating the IGF system; and (4) regulating fiber formation though DNA methylation. Conversely, excess maternal dietary protein appears to only have minor effects on early fetal muscle formation, although excess maternal dietary protein up-regulates the genes related to muscle fiber formation. Unexpectedly, little evidence exists in the literature that demonstrates maternal protein-deficient diets increase adiposity in porcine neonates. Further, the apparent lack of effect of maternal protein-deficiency on increased fat deposition in porcine neonates is inconsistent with gene expression in the neonates. It is possible, however, that the up-regulation of these functional genes might contribute to fat accumulation in the finishing period. Clearly, further research is needed to more clearly determine the effects of maternal protein-deficient diets on fat deposition in porcine fetuses and neonates. Sufficient maternal dietary protein can enhance porcine milk protein and fat concentration. L-arginine and BCAAs appear to increase protein synthesis in porcine mammary gland epithelial cells. However, research reports evaluating L-arginine and BCAAs in sows are limited and results are inconsistent. Currently, reports on maternal supplementation of functional amino acids (e.g., L-leucine and L-arginine) on fetal programing are limited. Future studies are needed to clarify mechanisms of effects of dietary amino acids in sows on their progeny. Furthermore, inconsistent results between genotype and phenotype indicate that non-coding RNA (miRNA, circular RNA) and DNA, and RNA methylation might participate in biological functions associated with fetal muscle fiber development during gestation and milk protein synthesis during lactation. Understanding the function of protein and amino acids in sows and effects on their offspring can provide rational approaches for regulation of piglet growth and further improvements in meat quality in the future. 

## Figures and Tables

**Table 1:** Effect of different maternal protein levels on early fetal muscle development. Upward-pointing arrow: “increase”; downward-pointing arrow: “decrease”; horizontal arrow: “no change”.

Animals	Experimental Design	Results	Conclusion	References
14 primiparous pure-bred Meishan gilt sbody weight: 36.1 ± 1.8 kg	Digestible energy 13 MJ/kg Control treatment: 12.2% crude protein (CP) (gestation) and 14% CP (lactation) Low protein treatment: 6.1% CP (gestation) and 6.9% CP (lactation)	Muscle protein synthesis: p70S6K and eIF4E ↓ Negative regulator of skeletal muscle development and growth ↑ Muscle weight: Longissimus dorsi muscles ↓	Maternal low protein diet throughout gestation and lactation causes retardation in muscle hypertrophy and protein synthesis	[28]
16 Landrace × Yorkshire crossbred sows in the second parity	Digestible energy:13 MJ/kg (gestation),14.39 MJ/kg (lactation) Control treatment: 15% (CP, gestation) and 18% CP (lactation) Low protein treatment: 7.5% (CP, gestation) and 9% CP (lactation)	Back fat thickness ↓ Restricted amino acid response (AAR) pathway: CHOP, IRE1α, PERK, ATF-6, XBP-1 and Bip, ATF4, and EIF2α↑ Autophagy-related genes: ATG7 and LC3 ↑	Maternal low protein diet throughout gestation and lactation causes offspring reduced adipogenesis and increased lipolysis	[33]
56 German Landrace gilts	Metabolic energy: 13.7 MJ/kg Adequate protein treatment: 12.1% CP High protein treatment: 30% CP; Low protein treatment, 6.5% CP	Total number of myofibers ↔ Myogenic regulatory factors in low protein treatment: MYOG, MRF4, IGF1, IGF1R ↓ Myogenic regulatory factors in high protein treatment: MYOD, MYOG, IGF1R, and IGFBP5 ↑	Moderate high or low maternal protein diets change gene expression but not the phenotype of skeletal muscle from porcine fetuses	[32]
47 pure German Landrace gilts	Metabolic energy: 13.7 ME/kg Adequate protein treatment: 12.1% CP High protein treatment: 30% CP; Low protein treatment, 6.5% CP	Percentage of muscle tissue in in HP in high protein treatment ↑ Primary and secondary myofibers in low protein diet treatment ↓ Subcutaneous adipose tissue mass in low protein diet treatment and high protein diet treatment ↓	Both limited and excess protein supply retards fetal growth, but only limited protein supply impairs myogenesis, persistently restricts muscle growth potential, and favors obesity at infancy.	[30]
24 primiparous German Landrace sows	Metabolic energy: 13.7 ME/kg Adequate protein treatment: 12.1% CP High protein treatment: 30% CP; Low protein treatment, 6.5% CP	Candidate genes of nutrient- dependent pre- and postnatal development: muscular expression of NCAPD2 (LP ↓), NCAPG (↔), NCAPH (LP ↓) Key genes of methionine metabolism: both HP and LP diet significant influence DNMT1 (HP ↑), DNMT3a (LP ↓) and MAT2B (HP ↑)	Maternal protein supply regulate condensin I subunit gene expression by methylation process and in turn may affect cell division in skeletal muscle tissue	[34]
